# Co-Expression of Chicken IL-2 and IL-7 Enhances the Immunogenicity and Protective Efficacy of a VP2-Expressing DNA Vaccine against IBDV in Chickens

**DOI:** 10.3390/v11050476

**Published:** 2019-05-24

**Authors:** Shanshan Huo, Jianlou Zhang, Jinghui Fan, Xing Wang, Fengyang Wu, Yuzhu Zuo, Fei Zhong

**Affiliations:** Laboratory of Molecular Virology and Immunology, College of Animal Science and Technology/College of Veterinary Medicine, Hebei Agricultural University; Hebei Engineering and Technology Research Center of Veterinary Biotechnology, Baoding 071000, Hebei, China; huoshanshan567@163.com (S.H.); zhangjianlouauh@163.com (J.Z.); Jinghui76@163.com (J.F.); wangxing4549@126.com (X.W.); fengyangwu2015@163.com (F.W.); zuoyuzhu@163.com (Y.Z.)

**Keywords:** chicken IL-2, chicken IL-7, mutual enhancement, immunogenicity, IBDV VP2 DNA vaccine

## Abstract

Chicken infectious bursal disease (IBD) is still incompletely controlled worldwide. Although IBD virus (IBDV) VP2 DNA vaccine was considered a safe vaccine for IBD prevention, the immunogenicity by itself remains poor, resulting in the failure of effectively protecting chickens from infection. We and others demonstrated that chicken IL-2 (chIL-2) and chIL-7 have the capacity to enhance the immunogenicity of the VP2 DNA vaccine. However, whether chIL-2 and chIL-7 can mutually enhance the immunogenicity of VP2 DNA vaccine and thereby augment the latter’s protection efficacy remains unknown. By using chIL-2/chIL-7 bicistronic gene vector to co-immunize the chickens together with the VP2 DNA vaccine, we now show that chIL-2 and chIL-7 significantly increased IBDV VP2-specific antibody titers, T cell proliferation, and IFN-γ production, resulting in the ultimate enhancement of vaccine-induced protection efficacy relative to that of chIL-2 or chIL-7 gene vectors alone. These results suggest that chIL-2 and chIL-7 can mutually enhance VP2 DNA vaccine’s efficacy, thereby establishing a concrete foundation for future optimization of IBDV VP2 DNA vaccine to prevent/treat chicken IBD.

## 1. Introduction

Infectious bursal disease (IBD) caused by IBD virus (IBDV) is a highly contagious and immunosuppressive disease affecting the poultry industry worldwide. IBDV specifically infects developing B cells in the bursa of Fabricius of young chickens, causing the destruction of the antibody-producing B cell precursors, with consequent lymphoid depletion of B cells and bursa atrophy, culminating in immunosuppression, vaccination failure, and susceptibility to other microbial infections [1].

IBDV, a member of the family of *Birnaviridae*, is a non-enveloped, double-stranded RNA virus, its genome contains two segments (A and B) [2]. The larger segment A (3.2 kb) has two open reading frames (ORFs), one of the ORFs encodes VP5 (17 kDa), a nonstructural protein being considered to be essential for viral release [3]; another ORF encodes a 110 kDa-precursor polyprotein (110 kDa), which is self-cleaved by its protease (VP4) to form three viral proteins: VP2 (48 kDa), VP3 (33–35 kDa) and VP4 (24 kDa) [4]. VP2 and VP3 are the major capsid proteins constituting 51% and 40% of the viral proteins, respectively [5]. Moreover, VP2 is also a major host-protective antigen of IBDV and contains major epitopes responsible for inducing viral neutralizing antibodies against IBDV [6]. The small segment B (2.9 kb) contains a single ORF encoding VP1 (90 kDa) which has RNA-dependent RNA polymerase activity [7].

IBD has not been effectively controlled so far although vaccination programs have been extensively implemented worldwide using live attenuated or inactivated IBDV vaccines, outbreaks of IBD still occur [8]. The live attenuated vaccine often exhibits strong immunogenicity, but is often associated with the emergence of highly virulent and variant strains, probably due to selection pressure from the administration of the live attenuated IBDV vaccine [9]. In addition, the live attenuated vaccine has often caused chicken immunosuppression and sub-clinical infection [10]. The inactivated IBDV vaccine is comparably safe but often displays relatively weak immunogenicity and low protective efficacy. Therefore, it is imperative to develop a safe and effective IBDV vaccine. In recent years, IBDV VP2-based DNA vaccine has been extensively studied as a potential candidate vaccine for the development of effective IBDV vaccine [11,12] because VP2 DNA vaccine can induce humoral and cellular immune responses [12], including neutralizing antibodies [6]. However, neither the full-length nor the truncated VP2 vaccine fully protected chickens from IBDV infection. 

Recently, some immunostimulatory cytokines are shown to be more effective biological adjuvants that enhance the immunogenicity of IBDV DNA vaccine. Chicken IL-2 [13], chIL-6 [14], chIL-18 [15], and chIFN-γ [16] have all been demonstrated to possess strong biological adjuvant activities that boost the efficacy of IBDV VP2 DNA vaccine. Our previous work showed that chIL-7 gene vector also had potent adjuvant activity and enhanced the immunogenicity of IBDV VP2 DNA vaccine [17]. Consistently, recombinant chIL-7 significantly boosted the immunogenicity of inactivated IBDV vaccine [18]. 

IL-2, -7, -9, -15, and -21 belong to the common γ-chain cytokine family [19] and have essential roles in the maintenance of immune homeostasis [20,21]. IL-2 drives the development and maturation of B cells, and also promotes T cell differentiation into antigen-specific effector and memory populations [21,22]. In contrast, IL-7 stimulates the differentiation of multipotent hematopoietic stem cells into lymphoid progenitor cells and proliferation of all cells in the lymphoid lineage (B cells, T cells, and natural killer (NK) cells) [23,24]. Our previous work showed that mouse IL-2 and IL-7 can mutually enhance the immunogenicity of ovalbumin (OVA) DNA vaccine [25]. However, whether their chicken counterparts behave similarly remains unclear. To bridge this knowledge gap, we used a bicistronic expression vector for synchronously expressing chIL-2 and chIL-7 as an adjuvant for IBDV VP2 DNA vaccine. We found that chIL-2 and chIL-7 can indeed mutually enhance the immunogenicity of IBDV VP2 DNA vaccine in IBD chickens, as shown by dramatically increased chicken humoral and cellular immune responses against the VP2 DNA vaccine and consequent augmented protective efficacy. Our results thus establish an essential basis for further improving the immune effect of the IBDV VP2 DNA vaccines.

## 2. Materials and Methods

### 2.1. Plasmids, Cells, Viruses, and Chickens

The pcDNA3.1A plasmid was from Invitrogen (Carlsbad, CA, USA). The pcDNA-chIL7, pcDNA-chIL7/H, pcDNA-VP2 (VP2 is an epitope in IBDV VP2 consisted of 122 amino acids) and pcDNA-VP2/H-IRES-chIL7/H and pcDNA-VP2-IRES-chIL7 plasmids were previously constructed in our laboratory [17]. Human embryonic kidney (HEK) 293T cells (ATCC CRL-11268) and UMNSAH/DF-1 chicken embryonic fibroblast cells (DF-1 cells) (ATCC CRL-12203) were purchased from ATCC (Manassas, VA, USA). A virulent IBDV strain (Harbin-1 strain) was kindly provided by Zandong Li of China Agricultural University. Specific pathogen–free (SPF) chickens were purchased from Jinan Sais Poultry Company (Jinan, China) and raised in an isolator in an environmental control room with a 12/12 h light/dark cycle. Animal experiments were approved by the Animal Ethics Committee of the Hebei Agricultural University (No. DW20170016) 

### 2.2. Vaccine, Antibodies, and Proteins

Attenuated live IBDV vaccine was from Ringpu (Baoding, China). Mouse anti-His monoclonal antibody and goat anti-mouse IgG-AP antibody were from Abcam (Cambridge, MA, USA). Recombinant IBDV VP2 protein was prepared in our laboratory as previously described [17].

### 2.3. Construction of chIL-2 Expression Vector and chIL-2/chIL-7 Bicistronic Expression Vectors

Chicken IL-2 cDNA with and without stop codons were amplified by RT-PCR from chicken spleen tissue using the following primers designed based on the chIL-2 sequence (GenBank: AF000631.1), in which *Eco*R I and *Xba* I sites (underlined) were introduced at the 5′ end of forward and reverse primers, respectively. chIL-2-F: 5′-CCGGAATTCACCATGA TGTGCAAAGTA CTGATC; chIL-2-R: 5′-GCTCTAGATTATTTTTGCAGATATCTCACA; chIL-2-F: 5′-CCGGAA TTCACCATGATGTGCAAAGTACTGATC; chIL-2-Rns: 5′-GCTCTAGATTTTTGCAGATATCTCA CAAAG. The amplified PCR products were cloned into pMD19-T vector and sequenced by Sangon Biotech (Shanghai, China), and then transferred into pcDNA3.1A plasmid to construct eukaryotic expression vector of chIL-2 gene fused and non-fused with His-tag, namely pcDNA-chIL2/H and pcDNA-chIL2. Chicken IL-2 and IL-7 bicistronic expression vectors were constructed by transferring chicken IL-2 gene from pcDNA-chIL2 plasmid into upstream of internal ribosome entry site (IRES) element in pcDNA-VP2/H-IRES-chIL7/H and pcDNA-VP2-IRES-chIL7 to replace VP2 gene to generate His-tag fused and non-fused chIL-2 and chIL-7 bicistronic expression vectors, pcDNA-chIL2/H-IRES-chIL7/H and pcDNA-chIL2-IRES-chIL7, respectively.

### 2.4. Expression in HEK293T Cells

Expression and purification of chIL-2 and chIL-7 in HEK293T cells were performed as previously described [17]. Briefly, HEK293T cells were transfected with pcDNA-chIL2/H, pcDNA-chIL7/H, and pcDNA-chIL2/H-IRES-chIL7/H plasmids (pcDNA3.1A plasmid as a negative control) using Lipofectamine 2000 transfection reagent according to the manufacturer’s protocol (Invitrogen, Carlsbad, CA, USA). The recombinant chIL-2 and chIL-7 in the culture medium were purified using Ni-NTA-agarose beads (Qiagen, Duesseldorf, Germany) and detected by western blot. 

### 2.5. Western Blot

The recombinant chIL-2 and chIL-7 were detected by western blot using mouse anti-His antibody followed by goat anti-mouse IgG-AP as previously described [17]. The blotting bands were visualized by staining with nitro-blue tetrazolium and 5-bromo-4-chloro-3′-indolyphosphate (NBT/BCIP) (Bio-Rad, Hercules, CA, USA).

### 2.6. Detections of VP2, chIL-2 and chIL-7 Expressions In Vivo by RT-PCR

In vivo expressions of chIL-2 and chIL-7 in injected chicken muscle tissues mediated by different vectors were detected with RT-PCR. One hundred mg of injected muscle tissue freshly collected at 2 days after immunization were homogenized. Total RNA was prepared using Trizol reagent (Invitrogen, Carlsbad, CA, USA). The cDNA was synthesized with M-MLV-reverse transcriptase. The VP2, chIL-2 and chIL-7 expressions were detected by PCR using following primers: VP2-F: 5′-AGCGGCCTGATCGTGTTCTTCC, VP2-R: 5′-GCGGCTCACCAGGCGGCAGT AG (153 bp); chIL-2-F: 5′-ACTCTGCAGTGTTACCTGGG, chIL-2-R: 5′-TGCATTCACTTCCGTGTGA (151 bp); chIL-7-F: 5′-CTGCCACTTCTCCTTGTTCTG, chIL-7-R: 5′-GACTAATGCTGCTTTCCTTCT AA (300 bp). Glyceraldehyde-3-phosphate dehydrogenase (GAPDH) served as an internal control using following primers: GAPDH-F: 5′-GTGGTGCTAAGCGTGTTATCATC, GAPDH-R: 5′-GGCAG CACCTCTGCCATC (269 bp).

### 2.7. IBDV Propagation and Titer Determination

Propagation and titration of IBDV were performed as previously described [26]. Briefly, four-week-old SPF white leghorn chickens were inoculated with 10^4^ median embryo infectious dose (EID_50_) virulent IBDV (0.2 mL per bird) by the nasal and eye-drop routes. Infected bursae were harvested at 3 days post-inoculation, homogenized with PBS, frozen (−70 °C) and thawed three times. Homogenates were clarified by centrifugation at 1000 g for 10 min at 4 °C. The virus titers in supernatant were determined using SPF chicken embryos and expressed as EID_50_. Non-virulent IBDV, a cell-adapted virus, was propagated in DF-1 chicken embryonic fibroblast cells. Virus titers were determined by Reed–Muench method and expressed as the 50% tissue culture infective dose (TCID_50_). 

### 2.8. Animal Experiment 

**Figure 1:**
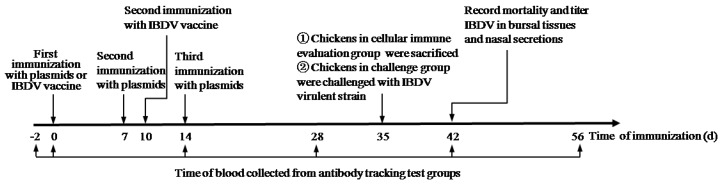
Time line of immunization.

A total of 300 SPF white leghorn chickens (21-day old) were randomly divided into 10 groups, 30 in each group (Table 1), and then each group was further divided into three groups, namely, an antibody tracking test group (8 birds), cellular immune evaluation group (8 birds), and challenge group (14 birds). The first group of chickens were not immunized and served as a negative control group. Chickens in group 10 were immunized orally with attenuated IBDV as positive control. Chickens in groups 2–9 were intramusculally injected with different immunization vectors (see Table 1). The immunized chickens were then boosted with the same vector and dose twice, 7 days apart. Chickens in group 10 were boosted with the same IBDV vaccine at the same titer at 10 days after the first immunization. At 2 days before the first immunization and at 0, 14, 28, 42, and 56 days after the first immunization (Figure 1), the blood samples were collected by the wing vein of the chicken from antibody tracking test group and the sera were isolated, and the titer of IBDV-specific antibodies were determined by enzyme-linked immunosorbent assay (ELISA). On the 35th day after immunization, chickens in the cellular immune evaluation group (8 birds) were euthanized, the splenic lymphocytes were aseptically separated by Ficoll density gradient centrifugation, cell proliferation index was determined by 3-(4,5-dimethylthiazol-2-yl)-2,5-diphenyltetrazolium bromide (MTT) assay, and IFN-γ and IL-4 expressions were determined by ELISA. At 35 d after immunization, chickens in the challenge group (14 birds) were challenged with 1 × 10^3^ ELD_50_ IBDV virulent strain (amplified using chicken embryo) by oral administration. Chicken mortality, bursa/body ratio (B/B ratio), bursa lesion score, and protective efficacy were evaluated by corresponding methods.

### 2.9. Detection of Serum Antibody Titers 

Antibody titers were determined by ELISA. Briefly, the 96-well plates were coated with 100 μL of VP2 protein (10 mg/mL) in coating buffer (0.05 M carbonate buffer, pH9.6) overnight at 4 °C and blocked with 3% non-fat milk in PBST. Then the plates were incubated with 100 μL of 2-fold serial diluted chicken sera at 4 °C for 1h. After washing with PBST, wells were incubated with 100 μL HRP-conjugated goat anti chicken IgG (Sigma, St. Louis, MO, USA) for 1 h at 37 °C. The freshly-prepared 3,3′,5,5′-Tetramethylbenzidine (TMB) solution (100 μL) was added for color development at 37 °C for 1 h. 50 μL of 2 M H_2_SO_4_ were then added to each well to stop the reaction and the values were read using a microplate reader at 450 nm.

### 2.10. Detection of Serum Neutralization Titers 

IBDV was amplified in DF-1 cells as previously described [27]. The titer of virus neutralization (VN) antibody was measured with the method described previously [28,29]. Briefly, serum was two-fold serially diluted (1:8–1:128) in a final volume of 75 μL of DMEM medium containing 10% (v/v) fetal bovine serum (FBS), 0.1 mM non-essential amino acids, 2 mM L-glutamine, 1 mM sodium pyruvate, 0.04 mM β-mercaptoethanol, 10 units/mL penicillin, 10 mg/mL streptomycin and mixed with 25 μL IBDV solution (2.5 × 10^2^ TCID_50_/mL). After incubation at 37 °C for 1 h, the mixture (100 μL) was used to infect chicken embryo fibroblast DF-1 cells cultured in 96-well plates. The cells were incubated at 37 °C and 5% CO_2_ for 5–7 d. The wells were scored for cytopathic effect (CPE). The VN antibody titer was defined as the reciprocal of the highest dilution that inhibited CPE in 50% of the wells.

### 2.11. Lymphocyte Proliferation Assay

MTT assay was used to measure lymphocyte proliferation [17]. Briefly, immunized chickens were euthanized at 35 days post-immunization and lymphocytes were isolated from spleens. Splenocytes were plated in 96-well plates at 2 × 10^6^ cells/well in RPMI-1640 medium with 10% FBS, and stimulated, in vitro, with concanavalin (Con A, 5 mg/mL, Sigma) as a positive control, and specific antigen VP2 protein (5 mg/mL) at 37 °C for 72 h. 20 μL MTT (5 mg/mL) were added to each well and incubated for 4 h. Cells were collected and incubated with 150 μL dimethyl sulfoxide (DMSO) to dissolve intracellular MTT. Supernatant was then transferred to another 96-well plate and the *OD*_450_ value was read in a microplate reader.

### 2.12. Detection of Cytokine Production

Spleen lymphocytes isolated from immunized chicken were stimulated with the VP2 protein as above. The IFN-γ and IL-4 levels in the culture medium were determined by ELISA using IFN-γ (SEA049Ga) and IL-4 (SEA077Ga) kits from Cloud-Clone Corp (Katy, TX, USA) and following the manufacturer’s instructions.

### 2.13. Viral Challenge Study

At 35 d post-immunization, the chickens in the challenge subgroups (14 birds in each group) were orally challenged with 1 × 10^3^ EID_50_ virulent IBDV. The challenged chickens were observed clinically for 8 d and mortalities were recorded. Chickens and bursae were weighed and B/B ratios were calculated by (bursal weight/body weight) × 1000. Bursal lesion scores were evaluated based on the histopathological severity of bursae [11]. Protection efficacy was defined by the number of chickens with histopathological BF lesion scores of 0 and 1 divided by the number of chickens in the group.

### 2.14. Statistics

The significance of differences between experimental groups was evaluated by one-way analysis of variance (ANOVA) with Dunnett’s post-comparison test for multiple groups and Student’s *t*-test was used for a single comparison of the two groups, respectively.

## 3. Results

### 3.1. Construction of chIL-2 and chIL-7 Expression Vectors 

**Figure 2:**
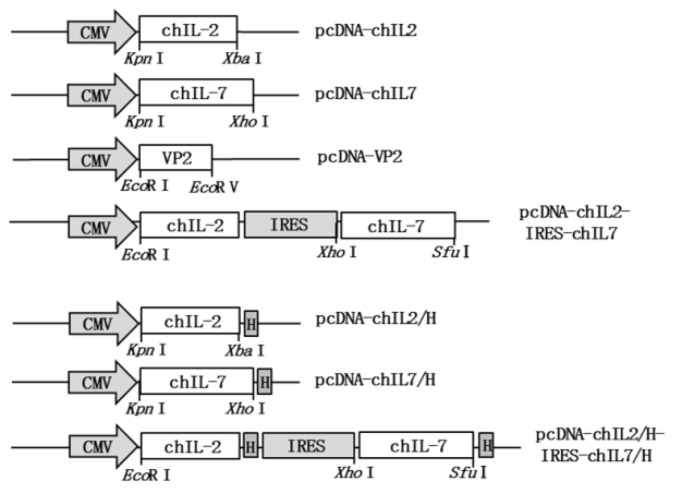
ChIL-2 and chIL-7 expression vector constructions. CMV, human cytomegalovirus promoter; H, 6× Histidine-tag; IRES, internal ribosome entry site.

To construct chIL-2 and chIL-7 expression vectors, the chIL-2 (432bp) gene was first amplified from chicken spleen by RT-PCR and then inserted into pcDNA3.1A plasmid to generate a His-tagged and non-tagged chIL-2 eukaryotic expression vectors, pcDNA-chIL2/H and pcDNA-chIL2. To construct chIL-2 and chIL-7 bicistronic expression vector, the VP2 gene in pcDNA-VP2/H-IRES-chIL7/H and pcDNA-VP2-IRES-chIL7 plasmids were substituted by chIL-2 gene to generate His-tag-fused and no-fused chIL-2 and chIL-7 bicistronic expression vectors: pcDNA-chIL2/H-IRES-chIL7/H and pcDNA-chIL2-IRES-chIL7, respectively (Figure 2). The chIL-7 gene vectors (His-tag fused and no-fused) were prepared previously in our laboratory [17]. The sequence of chIL-2 gene amplified in this study was identified to be consistent with that in GenBank (GenBank: AF000631.1) and the inserting sites of the genes in expression vectors were identified to be correct by restriction analysis and sequencing.

### 3.2. In Vitro and In Vivo Expression of chIL-2 and chIL-7 

**Figure 3:**
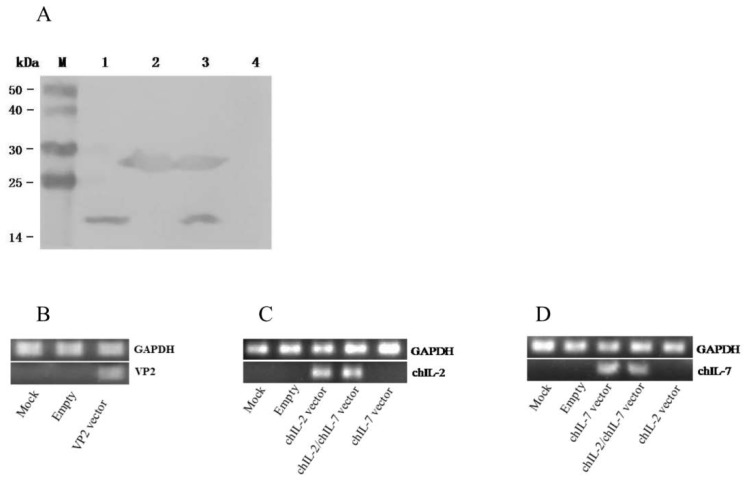
Detection of chIL-2 and chIL-7 expressions in vitro and in vivo. (**A**) Western blot for detection of chIL-2 and chIL-7 in the culture media of the vector-transfected HEK293T cells: M, prestained protein markers; Lane 1–4, transfected with pcDNA-chIL2/H, pcDNA-chIL7/H, pcDNA-chIL2/H-IRES-chIL7/H and pcDNA3.1A empty vector, respectively. (**B**–**D**) RT-PCR detection of VP2, chIL-2, and chIL-7 mRNA in the vector-injected chicken muscle tissues: GAPDH, glyceraldehyde-3-phosphate dehydrogenase as a house-keeping gene; Mock, untreated; Empty, injected with pcDNA3.1A plasmid; VP2 vector, injected with pcDNA-VP2 vector; chIL-2 vector, injected with pcDNA-chIL2 vector; chIL-2/chIL7 vector, injected with pcDNA-chIL2-IRES-chIL7 vector; chIL-7 vector, injected with pcDNA-chIL7 vector.

To determine whether the chIL-2 and chIL-7 vectors can mediate chIL-2 and chIL-7 expressions in eukaryotic cells in a secretory manner, we first tested the vector-mediated chIL-2 and chIL-7 expression in vitro. The HEK293T cells were transfected with His-tag-fused chIL-2 (pcDNA-chIL2/H) and chIL-7 (pcDNA-chIL7/H) gene vectors and pcDNA-chIL2/H-IRES-chIL7/H (pcDNA3.1A empty vector was transfected under the same conditions as a negative control). The expressed chIL-2 and chIL-7 in the culture media were detected by immunoblotting using anti-His-tag antibody. As shown in Figure 3A, the specific protein band of chIL-2 (about 15 kDa) and chIL-7 (about 25 kDa) were detected in the culture media from all three related vectors, indicating that the constructed vectors, either their individual or bicistronic vector, could mediate the corresponding gene expressions in a secretory manner in vitro. 

To determine whether the chIL-2 and chIL-7 vectors can mediate their gene expressions in vivo, we used RT-PCR method to detect chIL-2 and chIL-7 expressions in vector-injected chicken muscle tissues at 2 d after immunization. Just like in vitro experiments, VP2, chIL-2, and chIL-7 expressions were detected in the muscle tissues (Figure 3B–D), indicating that constructed expression vectors can mediate their corresponding gene expressions in vivo. 

### 3.3. ChckenIL-2 and chIL-7 Genes Mutually Enhance Humoral Immune Response to IBDV VP2 DNA Vaccine in Immunized Chickens 

**Figure 4:**
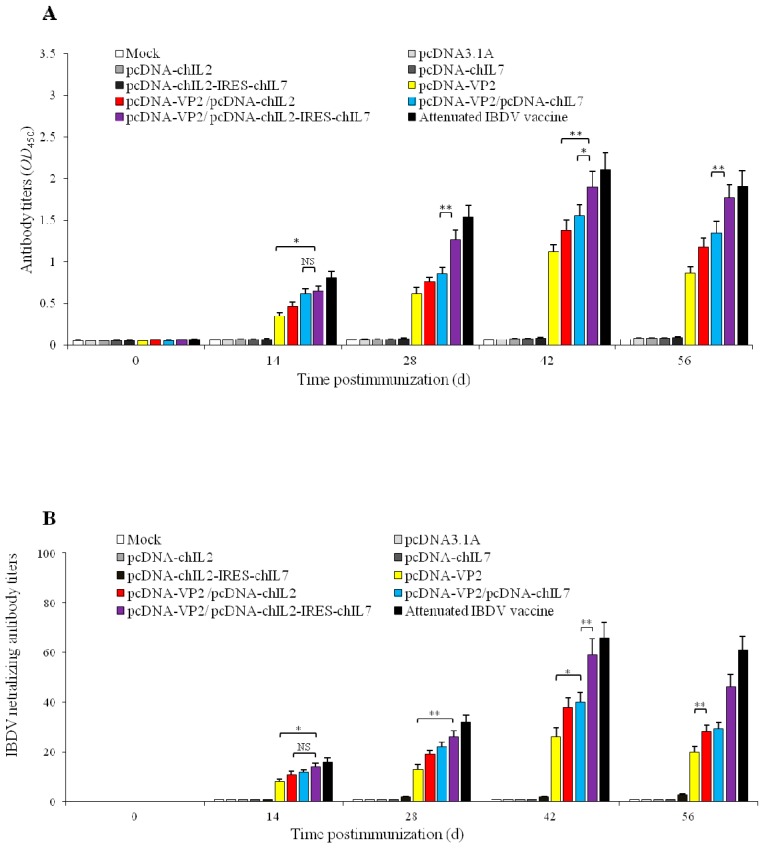
Antibody titers in immunized chicken and viral neutralization test. (**A**) IBDV antibody titers in the chickens of different groups at different time post immunization measured by ELISA. (**B**) IBDV neutralizing antibody titers in the chickens measured by viral neutralization test. Values are expressed as mean ± SD. ** *p* < 0.01, * *p* < 0.05, NS, no significance.

To investigate whether chIL-2 and chIL-7 genes have the mutual enhancement on humoral immune response to the VP2 DNA vaccine in chickens, we co-immunized the SPF chickens with different plasmids (Table 1) and measured the antibody titers at the different time post immunization by ELISA (Figure 4A). VP2-specific antibodies were detectable from day 14 and peaked on day 42 post-immunization, the titers were significantly increased in all immunized chickens. Importantly, the antibody titers in pcDNA-VP2/pcDNA-chIL2-IRES-chIL7 co-immunized chickens were significantly higher than that of either pcDNA-VP2/pcDNA-chIL2 (*p* < 0.01) or pcDNA-VP2/pcDNA-chIL7 co-immunized chickens (*p* < 0.01) (Figure 4A). Furthermore, the high-level neutralizing antibody against IBDV was also detected in co-immunized chickens with pcDNA-VP2/pcDNA-chIL2-IRES-chIL7 plasmids (Figure 4B). All above results indicate that both chIL-2 and chIL-7 enhance IBDV VP2 DNA vaccine immunogenicity, and co-administration of chIL-2/chIL-7 genes with IBDV VP2 DNA vaccine resulted in a significant increase in IBDV-specific antibody titers compared with separate administration, indicating that chIL-2 and chIL-7 possess mutual enhancement properties on chicken humoral immune response against IBDV.

### 3.4. Chicken IL-2 and chIL-7 Genes Mutually Enhance VP2 DNA Vaccine-Induced Cellular Immune Response in Immunized Chickens 

**Figure 5:**
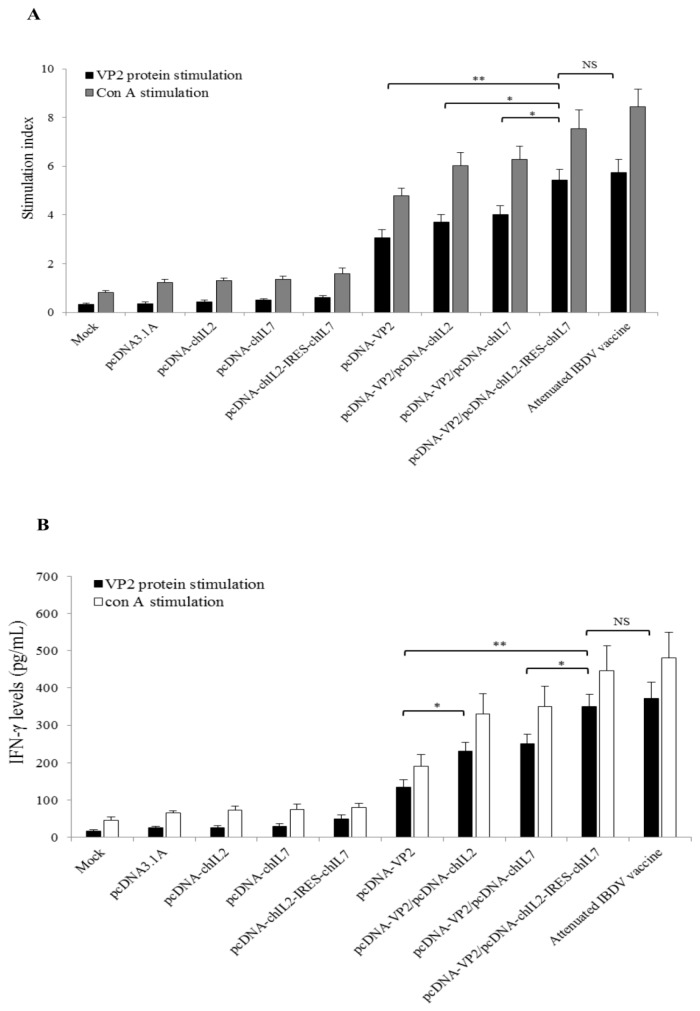

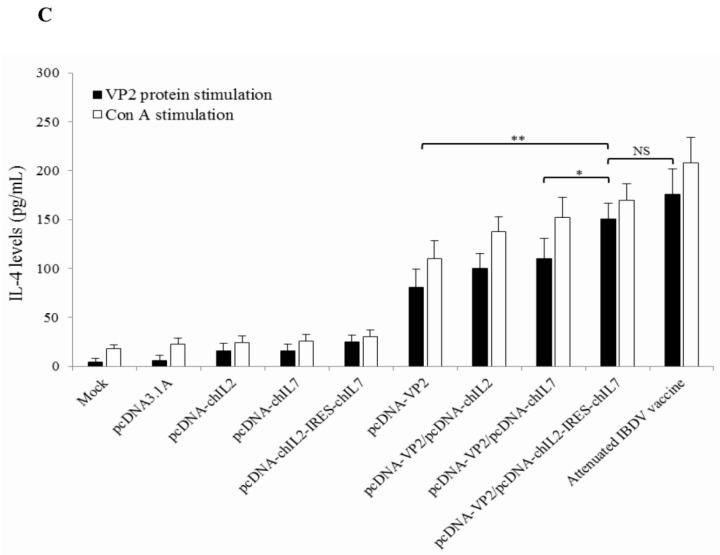
Lymphocyte proliferation and cytokine production in immunized chickens. (**A**) Lymphocytes were isolated from individual immunized chickens at 35 d post-immunization and re-stimulated with VP2 protein for 72 h or with Con A for 2 h in vitro. The stimulation indexes for each group immunized with different vectors were measured by MTT assay. (**B**) and (**C**) IFN-γ and IL-4 concentrations in culture media of lymphocytes from different immunized chickens were measured by ELISA. Values are expressed as mean ± SD. ** *p* < 0.01, * *p* < 0.05, NS, no significance.

To analyze whether chIL-2 and chIL-7 genes have the mutual enhancement on IBDV VP2 DNA vaccine-induced chicken cellular immune responses, the lymphocyte proliferation and cytokine production of immunized chickens were analyzed. Lymphocytes were isolated from spleen of the immunized chicken at 35 d post-immunization and stimulated with the VP2 protein (or Con A as a positive control). The cell proliferation was measured with MTT method and showed that the lymphocyte stimulation indexes (SI) of pcDNA-VP2/pcDNA-chIL2-IRES-chIL7 co-immunized and IBDV vaccine-immunized chickens were significantly higher than those of pcDNA-VP2 immunized (*p* < 0.01), pcDNA-VP2/pcDNA-chIL2 co-immunized, and pcDNA-VP2/pcDNA-chIL7 co-immunized chickens (*p* < 0.05) (Figure 5A). These results indicate that chIL-2 and chIL-7 have mutual enhancement on VP2 DNA vaccine-induced lymphocyte proliferation. To further test their mutual enhancement on cellular immune responses, IFN-γ and IL-4 expressions in spleen lymphocytes from the different immunized chickens were measured by ELISA after stimulation with the VP2 protein in vitro. Results showed that the IFN-γ and IL-4 levels in culture medium of lymphocytes from chIL-2/chIL-7 gene co-immunized chickens significantly higher than those from chIL-2 or chIL-7 gene co-immunized chickens (Figure 5B,C). Those above results indicate that chIL-2 and IL-7 have mutual enhancement on IBDV VP2 DNA vaccine-induced chicken cellular immune responses.

### 3.5. Chicken IL-2 and chIL-7 Genes Mutually Improve the Protective Efficacy of VP2 DNA Vacccine-Immunized Chickens against Virulent IBDV Challenge 

**Figure 6:**
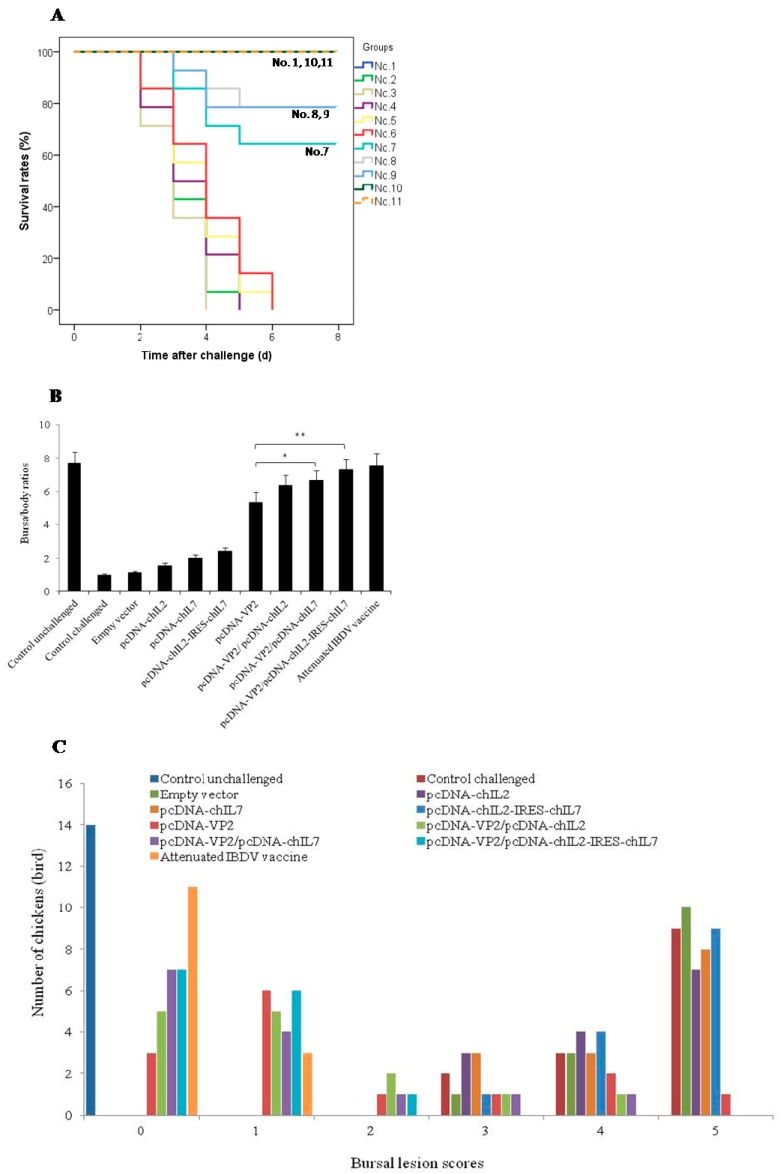

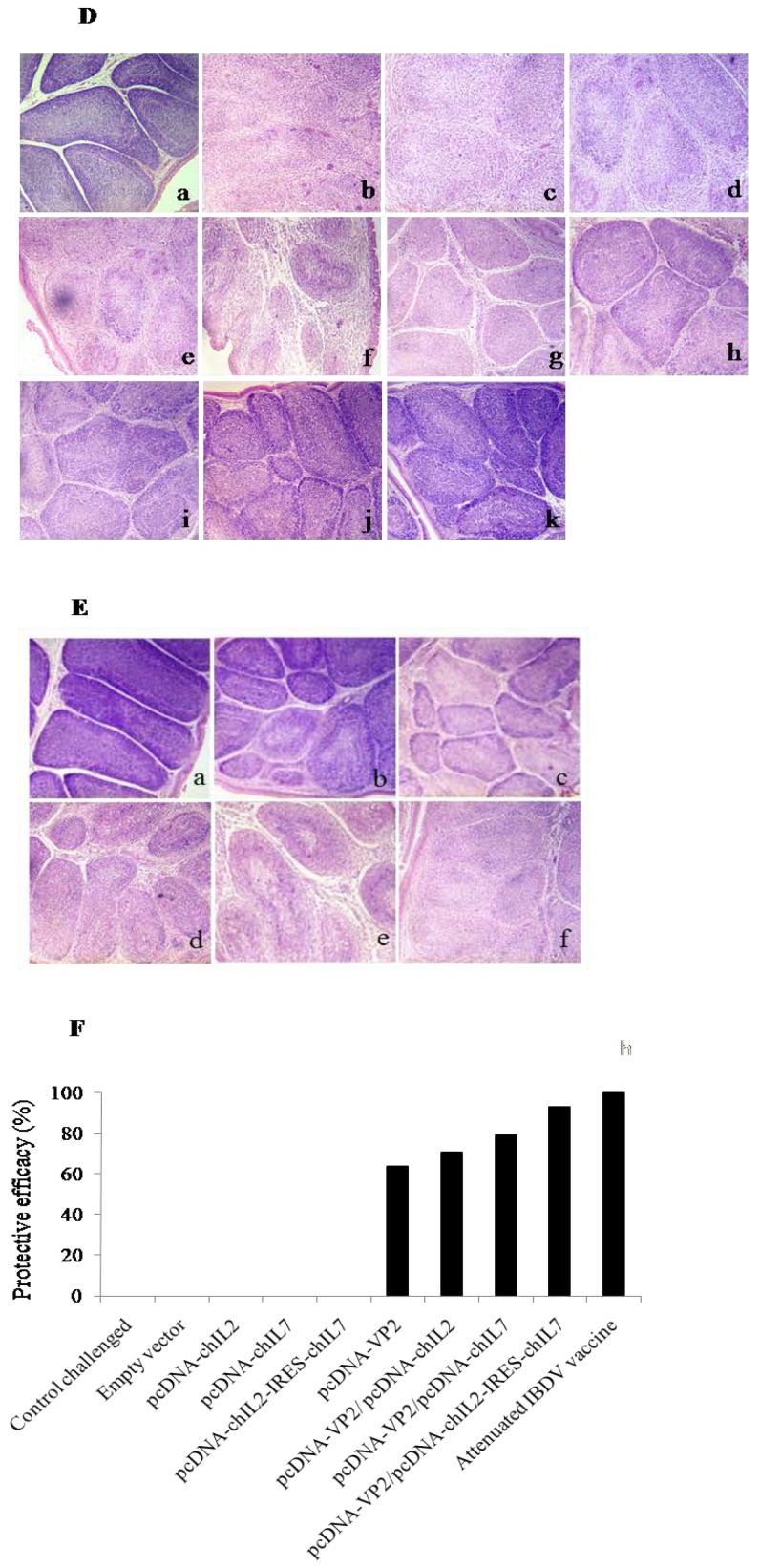

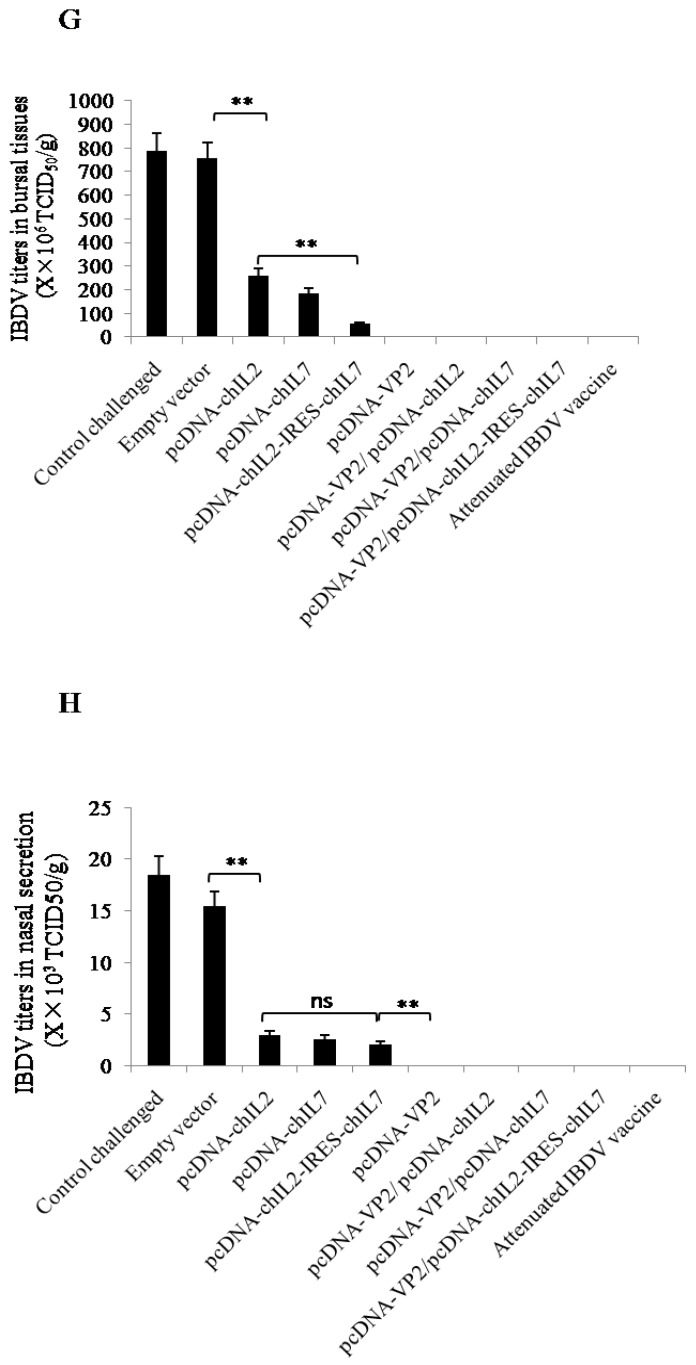
Protective effects of different DNA vaccines on IBDV-challenged chickens. (**A**) Survival rates, analyzed with the Kaplan–Meier survival curve. (**B**) Bursal body ratio, calculated by (bursal weight / body weight) × 1000 and presented as the mean ± SD from each group. (**C**) Bursal lesion scores, disigned from 0 to 5 according to the severity of bursal involvement at time of euthanasia (0: no lesion; 1: slight change, 2: scattered or partial bursal damage, 3: 50% or less follicle damage, 4: 51–75% follicle damage and 5: 76–100% bursal damage). (**D**) Histopathological BF lesion in different groups. a–k, control unchallenged, control challenged, empty vector, pcDNA-chIL-2, pcDNA-chIL -7, pcdNA-chIL-2-IRES-chIL-7, pcDNA-VP2, pcDNA-VP2/pcDNA-chIL-2, pcDNA-VP2/pcDNA- chIL-7, pcDNA-VP2/pcDNA-chIL-2-IRES-chIL-7, and attenuated IBDV vaccine, respectively. (**E**) Bursal lesion score criteria based on bursal histopathological characteristics. a–e, bursal lesion score 0, 1, 2, 3, 4, and 5, respectively. (**F**) Protective efficacy, defined by the number of chickens with histopathlogical BF lesion score 0 and 1/the number of chickens. (**G**) and (**H**) IBDV titers in bursal tissues and nasal secretion, respectively. Values are expressed as mean ± SD. ** *p* < 0.01, * *p* < 0.05, NS, no significance.

To evaluate the protective efficacy of VP2 DNA vaccine in immunized chickens co-administrated with the different chIL-2 and chIL-7 gene vectors, the co-immunized chickens in the challenge subgroups (except for group 1) were challenged with virulent IBDV. Clinical symptoms were observed and survival rates were recorded (Figure 6A) after the challenge. B/B ratios (Figure 6B), bursal lesion scores (Figure 6C,D) (based on bursal histopathological characteristics in Figure 6E), and protective efficacy (Figure 6F) were analyzed. IBDV titers in bursal tissues and nasal secretions are measured (Figure 6G,H). During the experimental period, chickens in group 1 (unchallenged) remained healthy and had normal sizes of bursae (0 score in Figure 6C), whereas those in other control groups (unimmunized, empty vector, and chIL-2 or chIL-7 vector immunized alone) showed typical clinical symptoms, and finally, the chickens successively died during the period of challenge. However, chickens immunized with VP2 DNA vector or VP2 vector plus chIL-2, chIL-7, or chIL-2/chIL-7 vector did not showed the typical clinical symptoms and high mortalities, the survival rates in pcDNA-VP2, pcDNA-VP2/pcDNA-chIL2, pcDNA-VP2/pcDNA-chIL7, and pcDNA-VP2/pcDNA-chIL2-IRES-chIL7-immunized chickens were 64, 79, 79, and 100%, respectively, significantly higher than those immunized with the control groups (Figure 6A). Importantly, the chIL-7/chIL-2 bicistronic vector-co-immunized chickens displayed higher survival rates compared with their individual vectors. Moreover, chickens immunized with pcDNA-VP2 plus pcDNA-chIL2-IRES-chIL7 had higher B/B ratios (Figure 6B), lower bursal lesion scores (Figure 6C), low IBDV titers in bursal tissues and nasal secretions than chickens immunized with pcDNA-VP2, pcDNA-VP2/pcDNA-chIL2, or pcDNA-VP2/pcDNA-chIL7 (Figure 6G,H). Protective efficacy of pcDNA-VP2/pcDNA-chIL2-IRES-chIL7-coimmunized chickens was 93% based on bursal lesion scores (Figure 6F), significantly higher than those of pcDNA-VP2 (64%), pcDNA-VP2/pcDNA-chIL2 (71%), and pcDNA-VP2/pcDNA-chIL7 (79%)-immunized chickens (Figure 6F). All results indicate that the chIL-2 and chIL-7 gene vectors have mutual enhancement on improvement of survival rates and protective efficacy of the chickens immunized with VP2 DNA vaccine.

## 4. Discussion

Relative to IBDV VP2 protein, IBDV VP2 DNA vaccine is widely considered to be a more effective vaccine since the latter is more potent at stimulating humoral and cellular immune responses that inhibit pathogen infection. Consistent with this notion, plasmid-based VP2 gene vector showed ~75% protective efficacy against virulent IBDV challenge [12]. Although the VP2 DNA vaccine has relatively high immunogenicity and an acceptable biosafety profile, it still falls short on conveying 100% protection from IBDV infection. Previous work has argued that both humoral and cellular immune responses may be needed for full protection. Several attempts that incorporate additional adjuvant(s) have been made to improve VP2 DNA vaccine’s immunogenicity, including cytokines [15,17,18,28,30], heat shock protein (HSP) [31], CpG-DON [32], and β-defensin-1 [33]. By far, cytokines appear to be the mostly studied biological adjuvants aiming at boosting the efficacy of IBDV VP2 DNA vaccines. For example, chicken common γ-chain cytokine family members (IL-2, IL-7, IL-9, IL-15, and IL-21) [13,30] have been extensively tested for their adjuvanticity for IBDV VP2 DNA vaccine.

Similar to its mammalian counterparts, chicken IL-2 can induce spleen T cell proliferation, increase the activity of NK cells [34], and enhance the immunogenicity of IBDV VP2 DNA vaccine [13,30]. In contrast, IL-7 is shown to stimulate B cell and T cell differentiation, proliferation, maturation, and maintenance [23,24]. Due to its potent immunostimulating property, IL-7 was used to treat immunosuppression diseases [35,36]. Additionally, IL-7 can also enhance vaccine immunogenicity by functioning as an adjuvant [37,38]. Our previous work demonstrated that the canine IL-7 gene can enhance the immunogenicity of canine parvovirus VP2 DNA vaccine [39,40]. Whether chicken IL-7 (chIL-7) gene behaves similarly has remained to be elucidated. Recently, we cloned the chIL-7 and characterized its biological function, and found that chIL-7 could induce B cell and T cell activation [41]. We also found that chIL-7 gene vector and recombinant chIL-7 could enhance the immunogenicities of IBDV VP2 DNA vaccine or an inactivated IBDV vaccine, respectively [17,18]. 

To further enhance the immunogenicity and thereby increase the protective efficacy of IBDV VP2 DNA vaccine, we used chIL-2 and chIL-7 bicistronic gene vector to further improve immune efficacy of the VP2 DNA vaccine. As expected, chIL-2 and chIL-7 mutually enhanced the immunogenicity of IBDV VP2 DNA vaccine, and the chIL-2/chIL-7 bicistronic gene vector also significantly increased VP2 DNA vaccine-induced humoral and cellular immune responses relative to those of chIL-2 or chIL-7 vector alone. Protection from infection was increased from ~80% in the chIL-2 or chIL-7 vector alone groups to 93% seen in the IL-2/IL-7 bicistronic vector group. These results shed light on the future optimization of DNA vaccine efficacy in the chicken IBD prevention and control. 

## 5. Conclusions

In summary, in this study we have demonstrated that chIL-2 and chIL-7 can mutually enhance the immunogenicity of the IBDV VP2 DNA vaccine. The IL-2/IL-7 bicistronic vector significantly increases chickens’ immune response elicited by the IBDV VP2 DNA vaccine, thereby resulting in significantly better protection against virulent IBDV challenge. This study opens up new ways (i.e., cytokines co-administration) to boost DNA vaccine for chicken IBD.

## Figures and Tables

**Table 1:** Chicken groups and plasmid dosages.

Group	Number of Chicken	Inoculum	Dose (µg)
1	30	Mock	0
2	30	pcDNA3.1A	200
3	30	pcDNA-chIL2	200
4	30	pcDNA-chIL7	200
5	30	pcDNA-chIL2-IRES-chIL7	200
6	30	pcDNA-VP2	200
7	30	pcDNA-VP2 + pcDNA-chIL2	200 + 200
8	30	pcDNA-VP2 + pcDNA-chIL7	200 + 200
9	30	pcDNA-VP2+ pcDNA-chIL2-IRES-chIL7	200 + 100
10	30	Attenuated IBDV vaccine (Ringpu)	2 × 10^3^ TCID_50_
